# Supplementary material for: Time-series transcriptome analysis identified differentially expressed genes in broiler chicken infected with mixed Eimeria species
Source: Front Genet. 2022 Aug 8;13:886781. doi: 10.3389/fgene.2022.886781 (PMC9393255; doi:10.3389/fgene.2022.886781)
Supplement: Supplementary file 2 [file DataSheet1.ZIP › 4dpi_KEGG.Gsea.1625100298584/KEGG_GNRH_SIGNALING_PATHWAY.html]

Details for gene set KEGG\_GNRH\_SIGNALING\_PATHWAY[GSEA]

|  || Dataset | TMM\_4dpi\_gct\_format\_4dpi\_gct\_format.Class\_4dpi.cls #PC\_versus\_NC.Class\_4dpi.cls #PC\_versus\_NC\_repos |
| Phenotype | Class\_4dpi.cls#PC\_versus\_NC\_repos |
| Upregulated in class | 1 |
| GeneSet | KEGG\_GNRH\_SIGNALING\_PATHWAY |
| Enrichment Score (ES) | 0.41355118 |
| Normalized Enrichment Score (NES) | 1.5160488 |
| Nominal p-value | 0.01779718 |
| FDR q-value | 0.26720253 |
| FWER p-Value | 0.9586 |
Table: GSEA Results Summary

  

Fig 1: Enrichment plot: KEGG\_GNRH\_SIGNALING\_PATHWAY      
 Profile of the Running ES Score & Positions of GeneSet Members on the Rank Ordered List

  

| SYMBOL | TITLE | RANK IN GENE LIST | RANK METRIC SCORE | RUNNING ES | CORE ENRICHMENT || 1 | PLA2G12B | na | 115 | 1.564 | 0.0586 | Yes |
| 2 | CACNA1D | na | 447 | 1.019 | 0.0753 | Yes |
| 3 | PLA2G6 | na | 448 | 1.018 | 0.1197 | Yes |
| 4 | PRKCD | na | 605 | 0.875 | 0.1448 | Yes |
| 5 | PTK2B | na | 713 | 0.810 | 0.1712 | Yes |
| 6 | ADCY8 | na | 750 | 0.789 | 0.2026 | Yes |
| 7 | PRKCA | na | 853 | 0.736 | 0.2262 | Yes |
| 8 | MAPK13 | na | 870 | 0.727 | 0.2566 | Yes |
| 9 | JUN | na | 912 | 0.705 | 0.2839 | Yes |
| 10 | PLCB1 | na | 1082 | 0.644 | 0.2978 | Yes |
| 11 | PLD1 | na | 1114 | 0.633 | 0.3229 | Yes |
| 12 | ADCY7 | na | 1147 | 0.620 | 0.3472 | Yes |
| 13 | CALM2 | na | 1272 | 0.583 | 0.3623 | Yes |
| 14 | KRAS | na | 1397 | 0.548 | 0.3758 | Yes |
| 15 | ITPR1 | na | 1663 | 0.481 | 0.3746 | Yes |
| 16 | MAP3K4 | na | 1809 | 0.454 | 0.3823 | Yes |
| 17 | MAPK12 | na | 2084 | 0.408 | 0.3771 | Yes |
| 18 | GRB2 | na | 2146 | 0.397 | 0.3893 | Yes |
| 19 | CAMK2G | na | 2166 | 0.394 | 0.4049 | Yes |
| 20 | PRKX | na | 2262 | 0.380 | 0.4136 | Yes |
| 21 | MAPK9 | na | 2786 | 0.310 | 0.3833 | No |
| 22 | GNRH1 | na | 3133 | 0.265 | 0.3659 | No |
| 23 | RAF1 | na | 3562 | 0.212 | 0.3393 | No |
| 24 | ADCY2 | na | 3608 | 0.206 | 0.3445 | No |
| 25 | CAMK2B | na | 3829 | 0.184 | 0.3341 | No |
| 26 | MMP2 | na | 3939 | 0.173 | 0.3326 | No |
| 27 | MAPK14 | na | 4107 | 0.159 | 0.3255 | No |
| 28 | MAP2K2 | na | 4498 | 0.124 | 0.2983 | No |
| 29 | SRC | na | 4500 | 0.124 | 0.3036 | No |
| 30 | MAPK3 | na | 4570 | 0.119 | 0.3030 | No |
| 31 | MAP2K1 | na | 4692 | 0.107 | 0.2976 | No |
| 32 | ITPR3 | na | 4698 | 0.106 | 0.3018 | No |
| 33 | EGFR | na | 4861 | 0.092 | 0.2922 | No |
| 34 | MAP2K4 | na | 5132 | 0.067 | 0.2726 | No |
| 35 | CDC42 | na | 5190 | 0.061 | 0.2705 | No |
| 36 | PLCB4 | na | 5226 | 0.059 | 0.2701 | No |
| 37 | MAP3K3 | na | 5360 | 0.048 | 0.2611 | No |
| 38 | MAPK10 | na | 5730 | 0.014 | 0.2308 | No |
| 39 | MAP2K3 | na | 5841 | 0.006 | 0.2219 | No |
| 40 | GNAS | na | 5856 | 0.005 | 0.2209 | No |
| 41 | HBEGF | na | 6054 | -0.011 | 0.2049 | No |
| 42 | CALM1 | na | 6078 | -0.013 | 0.2036 | No |
| 43 | ITPR2 | na | 6378 | -0.037 | 0.1801 | No |
| 44 | ADCY3 | na | 6636 | -0.057 | 0.1611 | No |
| 45 | PLCB2 | na | 6817 | -0.072 | 0.1492 | No |
| 46 | MAPK8 | na | 6831 | -0.073 | 0.1513 | No |
| 47 | CACNA1C | na | 6906 | -0.080 | 0.1486 | No |
| 48 | PRKACB | na | 7013 | -0.090 | 0.1436 | No |
| 49 | GNA11 | na | 7158 | -0.104 | 0.1361 | No |
| 50 | PLA2G4A | na | 7388 | -0.126 | 0.1224 | No |
| 51 | NRAS | na | 7621 | -0.146 | 0.1094 | No |
| 52 | MAP2K6 | na | 7919 | -0.172 | 0.0920 | No |
| 53 | MAP3K1 | na | 8024 | -0.182 | 0.0912 | No |
| 54 | GNAQ | na | 8151 | -0.193 | 0.0890 | No |
| 55 | ADCY6 | na | 8259 | -0.205 | 0.0890 | No |
| 56 | PLA2G4B | na | 8878 | -0.271 | 0.0491 | No |
| 57 | ADCY9 | na | 8880 | -0.271 | 0.0608 | No |
| 58 | SOS2 | na | 8925 | -0.276 | 0.0692 | No |
| 59 | CAMK2D | na | 8964 | -0.280 | 0.0782 | No |
| 60 | HRAS | na | 9244 | -0.315 | 0.0686 | No |
| 61 | ATF4 | na | 9591 | -0.361 | 0.0554 | No |
| 62 | MAPK11 | na | 10200 | -0.444 | 0.0238 | No |
| 63 | PRKCB | na | 10600 | -0.519 | 0.0131 | No |
| 64 | PLA2G2E | na | 11225 | -0.673 | -0.0098 | No |
| 65 | PLA2G5 | na | 11226 | -0.673 | 0.0195 | No |
| 66 | SOS1 | na | 11780 | -1.055 | 0.0193 | No |
Table: GSEA details [plain text format]

  

Fig 2: KEGG\_GNRH\_SIGNALING\_PATHWAY      
 Blue-Pink O' Gram in the Space of the Analyzed GeneSet

  

Fig 3: KEGG\_GNRH\_SIGNALING\_PATHWAY: Random ES distribution      
 Gene set null distribution of ES for **KEGG\_GNRH\_SIGNALING\_PATHWAY**
